# Gastric Peroral Endoscopic Tunneled Stricturotomy for Post‐Sleeve Gastrectomy Stenosis: Case Series and Literature Review

**DOI:** 10.1002/deo2.70290

**Published:** 2026-02-14

**Authors:** Shiv R. Patel, Christopher J. Farid, Thomas Shin, Alexander J. Podboy

**Affiliations:** ^1^ Department of Medicine University of Virginia Health System Charlottesville Virginia USA; ^2^ Division of Gastroenterology and Hepatology University of Virginia Health System Charlottesville Virginia USA; ^3^ Department of Surgery University of Virginia Health System Charlottesville Virginia USA

**Keywords:** balloon dilation, gastric stenosis, G‐POEM, sleeve gastrectomy, stricturotomy

## Abstract

Gastric peroral endoscopic tunneled stricturotomy (G‐POETS) has emerged as a novel procedure to treat post‐sleeve gastrectomy stenosis. We present a case series highlighting the use of G‐POETS to treat sleeve stenosis. Clinical success was assessed using the Dakkak‐Bennett Dysphagia Score and the Gastroparesis Cardinal Symptom Index. Clinical success was observed in five out of six patients. No adverse events occurred due to G‐POETS. The first literature review on the use of G‐POETS for sleeve stenosis was performed.

## 1 Introduction

Sleeve gastrectomy is the most performed bariatric surgery in the United States. Sleeve stenosis develops in up to four percent of cases and remains a challenging complication [1]. It typically arises at the incisura angularis from narrowing or torsion of the gastric sleeve. Narrowing occurs from fibrotic strictures in the layers of the stomach wall, including involvement of the muscular layer. Torsion occurs due to staple line geometry or unequal traction from the sleeve. Stenosis is categorized into two primary types: classic and helical. Classic stenosis involves luminal narrowing that is straight and symmetric, running parallel to the gastric axis without twisting. Helical stenosis has a spiral or twisted morphology, with asymmetric stenosis.

Patients can present with nausea, vomiting, dysphagia, or abdominal pain. Symptoms may manifest within weeks of surgery or months to years later as scarring and tissue remodeling occur in the area of stenosis [2]. Because of its variable timing and nonspecific symptoms, diagnosis is often delayed.

Management of sleeve stenosis is difficult as no standardized guidelines exist. Endoscopic balloon dilation (EBD) has traditionally served as the first line. However, response rates decline with longer follow‐up, and some patients are refractory. For these patients, revisional surgery, commonly conversion to Roux‐en‐Y gastric bypass (RYGB), is considered but carries increased morbidity and technical complexity. Endoscopic stenting of the gastric lumen is another technique that has been used. Gastric peroral endoscopic tunneled stricturotomy (G‐POETS) has emerged as an intervention that enables targeted myotomy of the stenotic segment. G‐POETS may be effective in resistant cases, as EBD mainly stretches the stomach wall, while G‐POETS uses full‐thickness dissection through the muscularis layer and serosa of the stomach.

We present six patients (mean age 59.5 ± 12.4 years), including four females (67%) and two males (33%), treated with G‐POETS for refractory sleeve stenosis (defined as failed initial EBD to 20 mm) from February 2024 to July 2025. G‐POETS was offered as part of clinical care. The initial EBD dilation diameter was based on institutional practices. Consent was obtained before G‐POETS. IRB approval, including category four exemption, was received for this retrospective review. All procedures were performed by a single provider. All consecutive patients who underwent this procedure for sleeve stenosis were included.

G‐POETS consisted of five steps: initial inspection, mucosotomy, submucosal tunneling, full‐thickness stricturotomy, and mucosal closure. Initial upper endoscopy was used to clear retained contents. A lifting solution (4–6 mL methylene blue + saline) was injected ∼5 cm proximal to the stenosis, followed by a 1–1.5 cm mucosotomy using a Hybrid triangle‐tip (TT) knife (20150‐260, ERBE) with EndoCut I current (50 W, effect 3; VIO 300D, ERBE). A submucosal tunnel was created toward the narrowing using the TT or I‐hybrid Endosurgical knives (20150‐260/261, ERBE) in spray or forced coagulation (50–80 W, effect 2), with repeated lifting injections for visualization. Submucosal vessels were coagulated with Olympus FD‐410LR graspers in soft coagulation mode (80 W, effect 3). Once the stenosis was reached, dissection extended 2–3 cm distally. A full‐thickness stricturotomy was then performed using EndoCut I current, continuing through the muscular layer to the serosa and extending beyond both margins of the stricture. The mucosotomy was closed with the Overstitch Endoscopic Suturing System with clips as needed (Figure S1).

Patients followed pre‐procedure NPO and received general anesthesia. Afterward, they were admitted for observation, received IV antibiotics, and remained NPO pending a Gastrografin study to rule out leakage. With normal imaging, they advanced to a liquid diet and were discharged on a 5‐day oral antibiotic course and twice‐daily PPIs for 2–3 months.

Baseline demographic, clinical, and pre‐procedural data were collected. Technical success was defined as completion of G‐POETS. Clinical success was defined as ≥1‐point reduction in the Gastroparesis Cardinal Symptom Index (GCSI) or Dakkak‐Bennett Dysphagia Score(DBS) at 2 months of follow‐up outpatient. This threshold represents a commonly adopted cutoff in the literature where these tools have been applied. GCSI was selected since it assesses symptoms across three domains: nausea/vomiting, early satiety/fullness, and bloating/distension [3]. DBS was selected due to dysphagia being a common symptom [4].

## 2 Case Report

**FIGURE 1:**
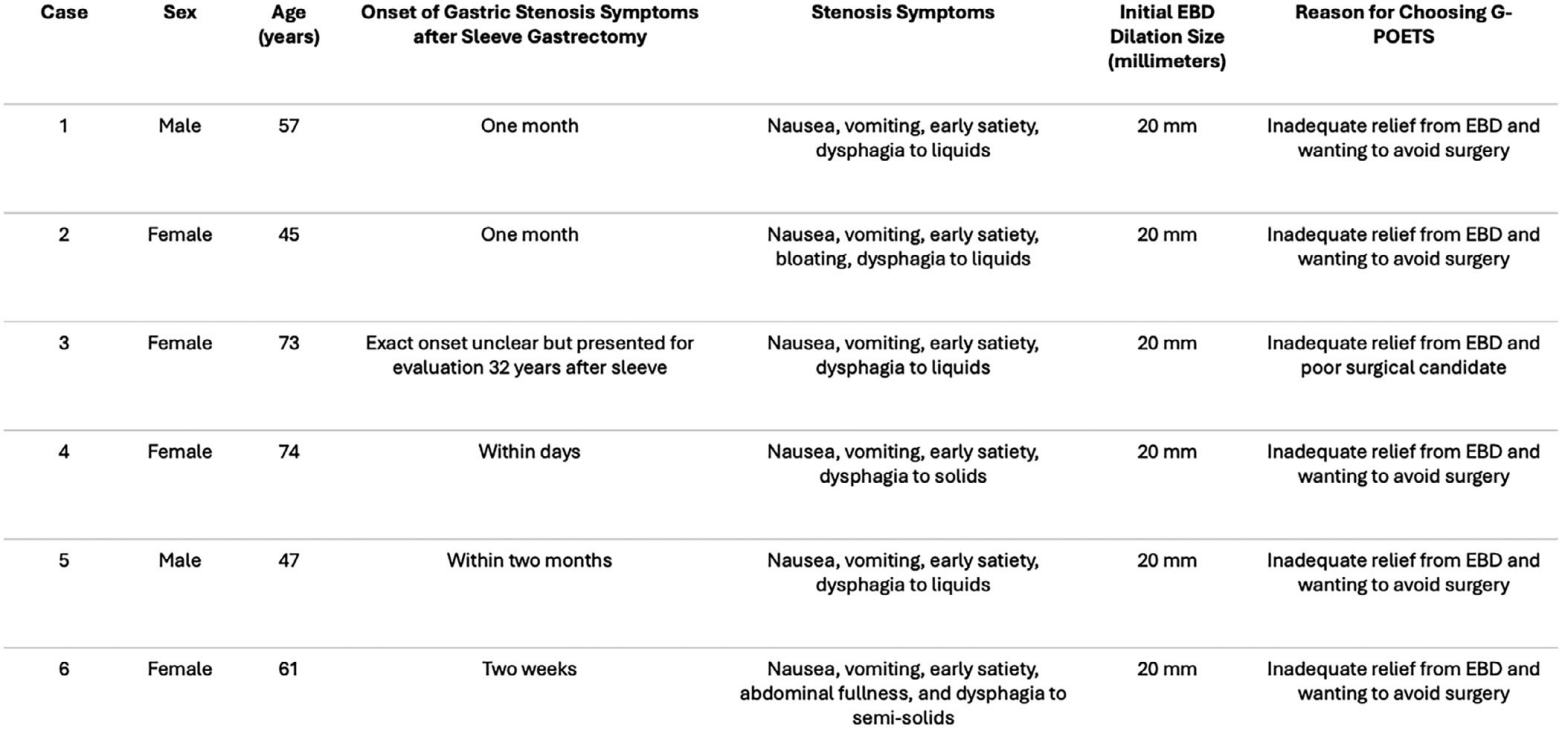
Baseline demographics, timing of symptom onset, symptoms, initial endoscopic balloon dilation (EBD) dilation size, and patient reasons for choosing gastric peroral endoscopic tunneled stricturotomy (G‐POETS).

Patients presented at variable times after sleeve gastrectomy with nausea, vomiting, early satiety, bloating, abdominal fullness, and dysphagia. All six patients initially underwent EBD. Four patients had endoscopically confirmed classic gastric stenosis, while two had endoscopically confirmed helical gastric stenosis. G‐POETS was offered to all the patients. Despite cases three and four having low initial symptom scores, they were offered G‐POETS because of impaired quality of life and desire for further intervention. All six patients subsequently proceeded with G‐POETS for one or more of the following reasons: they no longer wanted to repeat EBD (including deeper dilation) due to inadequate relief, preferred a nonsurgical approach, or were deemed poor surgical candidates (Figure 1).

**FIGURE 2:**
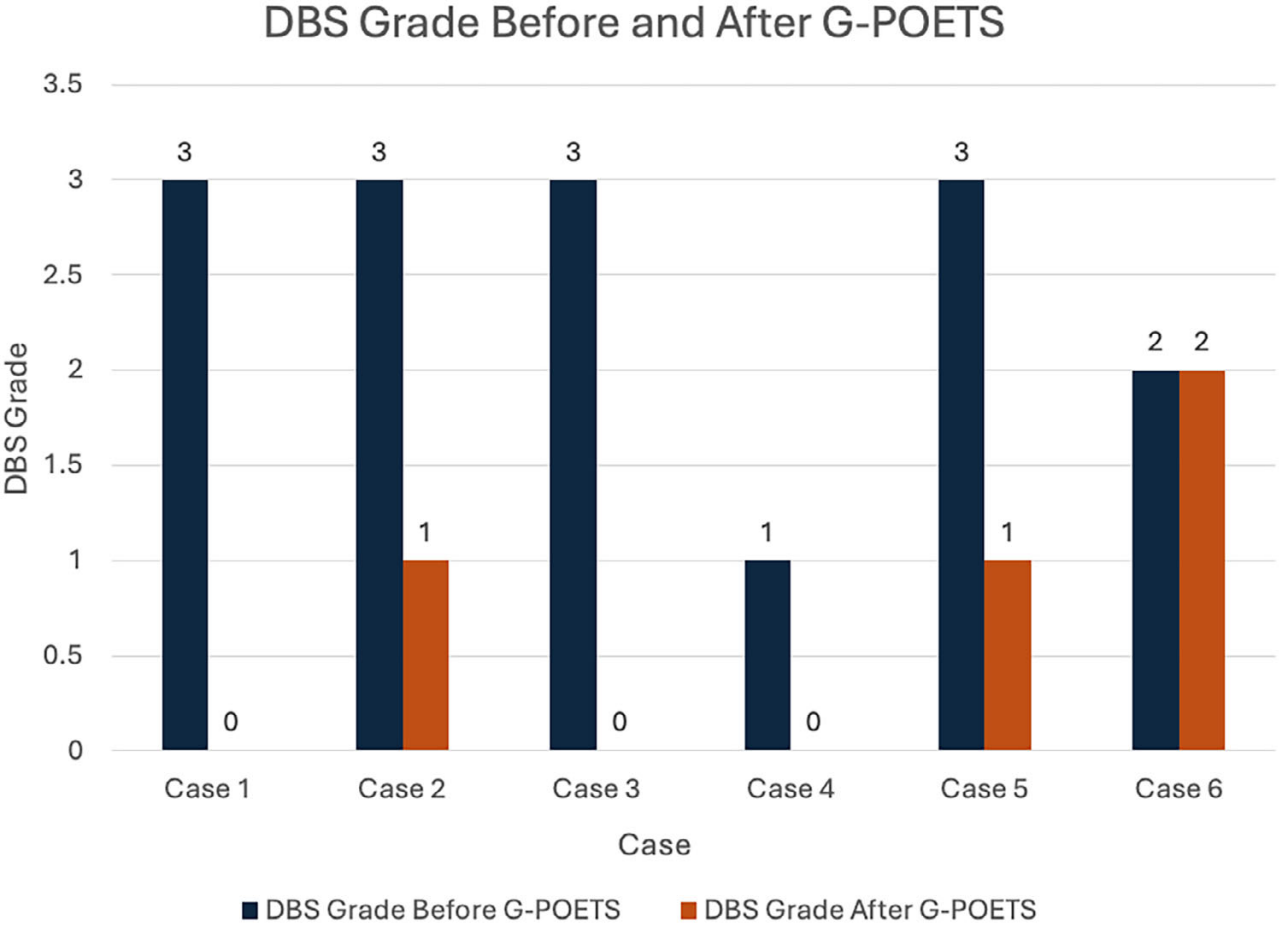
Comparison of Dakkak‐Bennett Dysphagia Score (DBS) at baseline and 2 months follow‐up.

**FIGURE 3:**
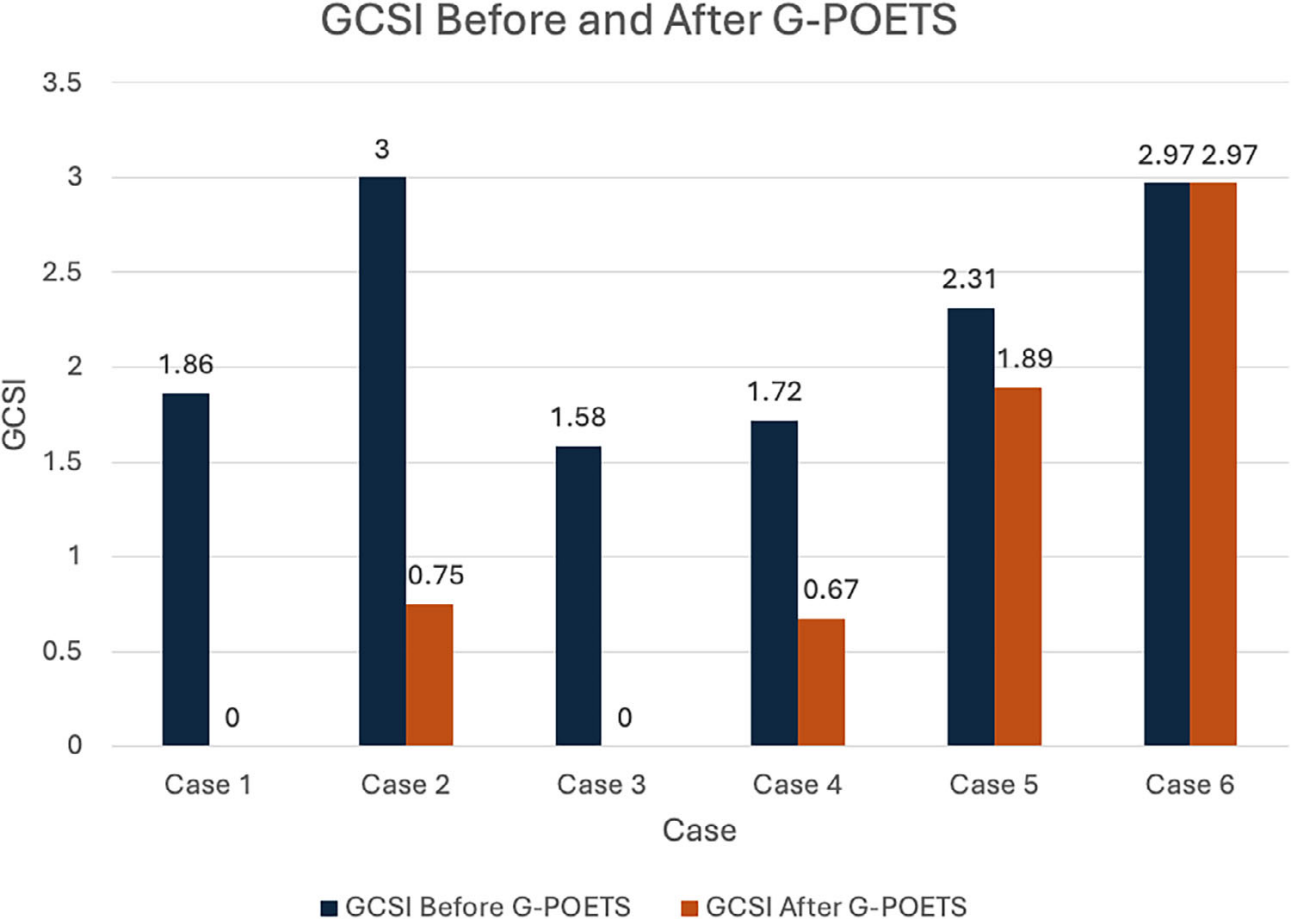
Comparison of Gastroparesis Cardinal Symptom Index (GCSI) at baseline and 2 months follow‐up.

All six patients underwent technically successful G‐POETS. No major complications occurred. Clinical success was achieved in 83% of patients. Despite case five requiring another EBD after G‐POETS, this patient was included as a clinical success due to a reduction in DBS (Figures 2 and 3).

## 3 Discussion

**FIGURE 4:**
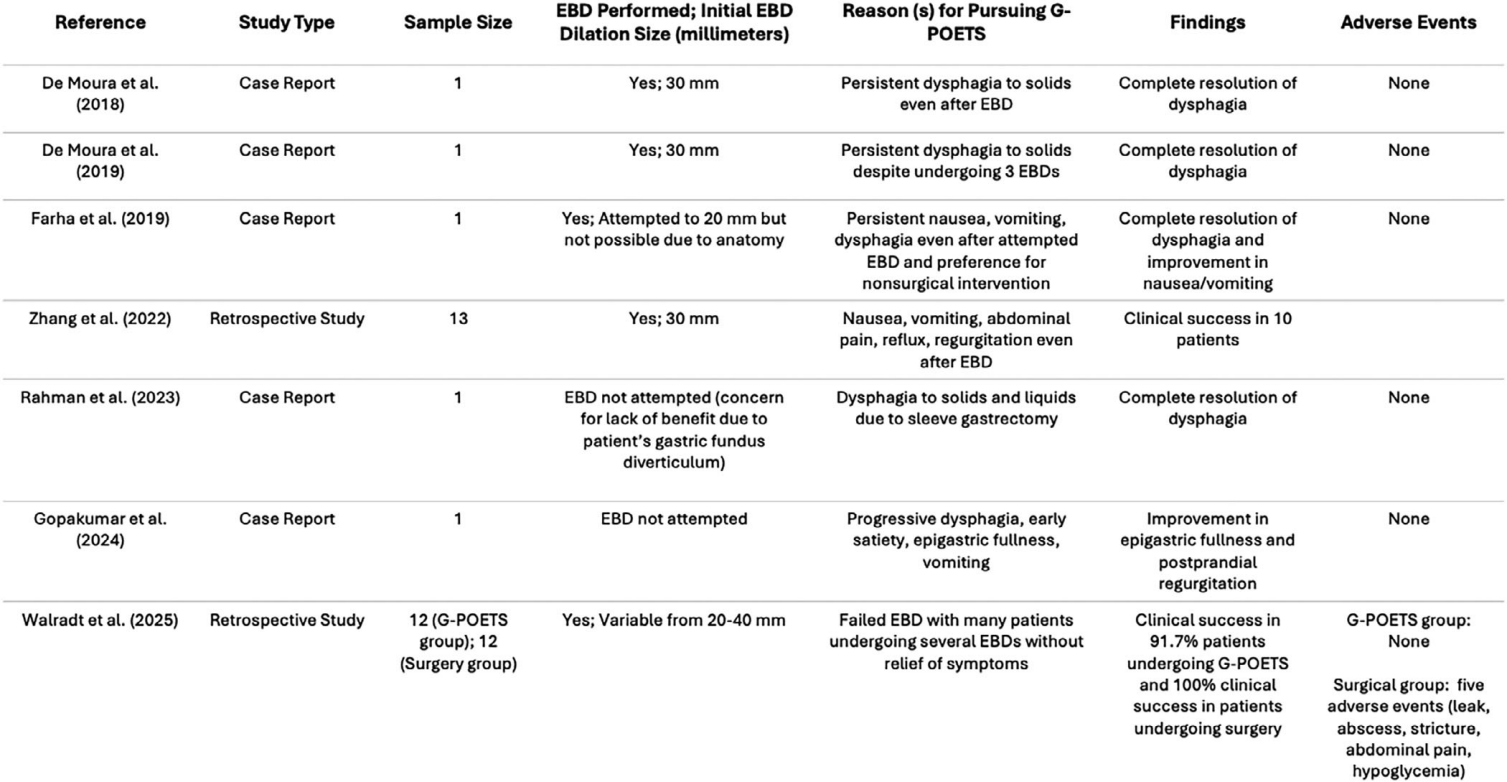
Summary of current literature published regarding the use of gastric peroral endoscopic tunneled stricturotomy (G‐POETS) for post‐sleeve gastrectomy stenosis.

We conducted a literature review using PubMed, ScienceDirect, and Scopus databases. Search terms included: gastric peroral endoscopic tunneled stricturotomy, gastric peroral endoscopic myotomy, endoscopic tunneled stricturotomy, sleeve gastrectomy, and sleeve stenosis. To our knowledge, we included all available reports that described this technique for sleeve stenosis, comprising five case reports and two retrospective studies. Terminology varied, with some referring to G‐POETS as “endoscopic tunneled stricturotomy” or “modified G‐POEM” (Figure 4).

De Moura et al. were the first to demonstrate the use of G‐POETS in two young patients with stenosis refractory to EBD. Differently, from our series, De Moura et al. attempted initial EBD to 30 mm instead of 20 mm, and patients still had poor relief. The two patients of De Moura et al. had symptoms within 1 month of gastrectomy [1, 5]. From De Moura et al., it can be derived that young patients with early symptoms and intervention can have resolution of dysphagia with G‐POETS. A similar conclusion can be taken from Rahman et al., who highlighted a 26‐year‐old female with complete resolution of dysphagia with later symptom onset at around 8 months [6]. Zhang et al. provided the hypothesis that G‐POETS may be less effective in patients with nonhelical stenosis. They believe that twisting in helical stenosis is more amenable to myotomy, which can manually unkink the narrowing [7]. Zhang et al. used GCSI without predefined objective thresholds for clinical success. From Farha et al. and Gopakumar et al., it can be synthesized that older patients and later symptom onset, particularly several years, have less favorable responses to G‐POETS [8, 9]. Walradt et al., in their retrospective matched cohort study, the first to compare G‐POETS to surgery, showed that although clinical success was greater in a surgical RYGB group compared to G‐POETS (100% vs 91.7%), it was not statistically significant. The rate of adverse events was in a statistically significant manner lower in G‐POETS compared to surgery (0% vs 41.7%). These findings suggest G‐POETS may be safer in patients with similar risk profiles, although the sample size was limited [10].

Rather than positioning G‐POETS as curative for all cases, the evidence indicates it should be incorporated into a treatment algorithm based on patient characteristics. While EBD applies a stretching force at the level of stenosis, G‐POETS directly disrupts stenosis by full‐thickness dissection, which can promote greater true luminal patency through structural remodeling rather than temporary stretching. G‐POETS may be less effective in older patients, with a later onset of symptoms, or non‐helical stenosis morphology. In our series, case six could have had clinical failure due to the patient having non‐helical stenosis and being older. Although no major complications related to G‐POETS occurred in the case series or literature, safety considerations include bleeding from damage to submucosal or muscular vessels, perforation from poor stenosis visualization, gastric motility dysfunction from over‐disruption of the stenotic segment, worsening scarring from permanent myotomy, and infection.

Our series is the first to evaluate G‐POETS using two validated symptom‐based scoring tools with GCSI and DBS, a new contribution to the literature. We hypothesize that G‐POETS may most benefit the symptom of dysphagia due to the vast improvement in DBS. We acknowledge that our reported outcomes reflect short‐term symptom improvement. Our small sample limits the generalizability of the findings. Symptom scoring and assessment are subjective, which may introduce bias. Additionally, this is the first literature review regarding this subject. G‐POETS offers an alternative when EBD is ineffective, a nonsurgical option is preferred, or revision surgery poses risk. Larger studies with longer follow‐up are needed to define its effectiveness compared to EBD and surgery.

## Author Contributions

**Shiv R. Patel**: completed design of manuscript, initial draft, data curation, literature review, visualization, revisions, and final draft review. **Christopher J. Farid**: revisions. **Thomas Shin**: performed final draft review and supervision. **Alexander J. Podboy**: conceptualization, performed serial and final draft review, supervision, and mentorship to the primary author.

## Funding

None

## Ethics Statement

Approval of retrospective research protocol by UVA Institutional Review Board (Study HSR210392).

## Conflicts of Interest

Thomas Shin, MD, PhD, is a Consultant for UBS and Intuitive Surgical. The other authors declare no conflicts of interest.

## Supporting information




**FIGURE S1**: Images highlighting the steps of the G‐POETS procedure. (a) Endoscopic identification of stricture. (b) Initial incision before insertion into the tunnel. (c) Stricture delineation. (d) Full‐thickness stricturotomy. (e) Submucosal tunnel closure. (f) Improvement in stricture opening.

## References

[1] E. G. H. De Moura , D. T. H. de Moura , and C. M. Sakai , “Endoscopic Tunneled Stricturotomy With Full‐Thickness Dissection in the Management of a Sleeve Gastrectomy Stenosis,” Obesity Surgery 29, no. 8 (2019): 2711–2712. 31140166 10.1007/s11695-019-03919-z

[2] M. Eguchi , R. Nogueira , C. A. Balthazar da Silveira , et al., “Endoscopic Management of Gastric Stenosis After Sleeve Gastrectomy: A Systematic Review and Single‐Arm Meta‐Analysis,” Surgical Endoscopy 39 (2025): 5324–5347. 40603612 10.1007/s00464-025-11843-w

[3] D. A. Revicki , A. M. Rentz , and D. Dubois , “Gastroparesis Cardinal Symptom Index (GCSI): Development and Validation of a Patient Reported Assessment of Severity of Gastroparesis Symptoms,” Quality of Life Research 13, no. 4 (2004): 833–844. 15129893 10.1023/B:QURE.0000021689.86296.e4

[4] M. Dakkak and J. R. Bennett , “A New Dysphagia Score With Objective Validation,” Journal of Clinical Gastroenterology 14, no. 2 (1992): 99–100, 10.1097/00004836-199203000-00004. 1556441

[5] D. T. H. De Moura , P. Jirapinyo , and H. Aihara , “Endoscopic Tunneled Stricturotomy in the Treatment of Stenosis After Sleeve Gastrectomy,” VideoGIE 4 (2018): 68–71. Published November 13, 2018. 30766946 10.1016/j.vgie.2018.09.013 PMC6362310

[6] S. H. Rahman and P. Kedia , “Modified Gastric‐Peroral Endoscopic Myotomy With Sleeve Release in a Case of Severe Gastric Sleeve Stenosis,” VideoGIE 9, no. 1 (2023): 8–11. Published September. 38261837 10.1016/j.vgie.2023.09.006 PMC10794108

[7] L. Y. Zhang , M. I. Canto , M. A. Schweitzer , et al., “Gastric per‐Oral Endoscopic Myotomy (G‐POEM) for the Treatment of Gastric Sleeve Stenosis: A Feasibility and Safety Study,” Endoscopy 54, no. 4 (2022): 376–381. 34225370 10.1055/a-1544-4923

[8] J. Farha , L. Fayad , A. Kadhim , et al., “Gastric Per‐Oral Endoscopic Myotomy (G‐POEM) for the Treatment of Gastric Stenosis Post‐Laparoscopic Sleeve Gastrectomy (LSG),” Obesity Surgery 29, no. 7 (2019): 2350–2354. 31001761 10.1007/s11695-019-03893-6

[9] H. Gopakumar and S. R. Puli , “Modified Peroral Endoscopic Myotomy for Non‐Helical‐Type Gastric Sleeve Stenosis After Laparoscopic Sleeve Gastrectomy,” Gastrointestinal Endoscopy 99, no. 2 (2024): 287–288. 37742774 10.1016/j.gie.2023.09.015

[10] T. Walradt , D. Szvarca , K. F. Schuster , et al., “Endoscopic Tunneled Stricturotomy versus Surgical Conversion to Roux‐en‐Y Gastric Bypass for the Management of Stenosis After Sleeve Gastrectomy,” Gastrointestinal Endoscopy 101, no. 5 (2025): S65–S66. 10.1016/j.gie.2025.08.012 40819677

