# Supplementary figures and images for: Gastric Peroral Endoscopic Tunneled Stricturotomy for Post‐Sleeve Gastrectomy Stenosis: Case Series and Literature Review
Source: DEN Open. 2026 Feb 14;6(1):e70290. doi: 10.1002/deo2.70290 (PMC12906293; doi:10.1002/deo2.70290)

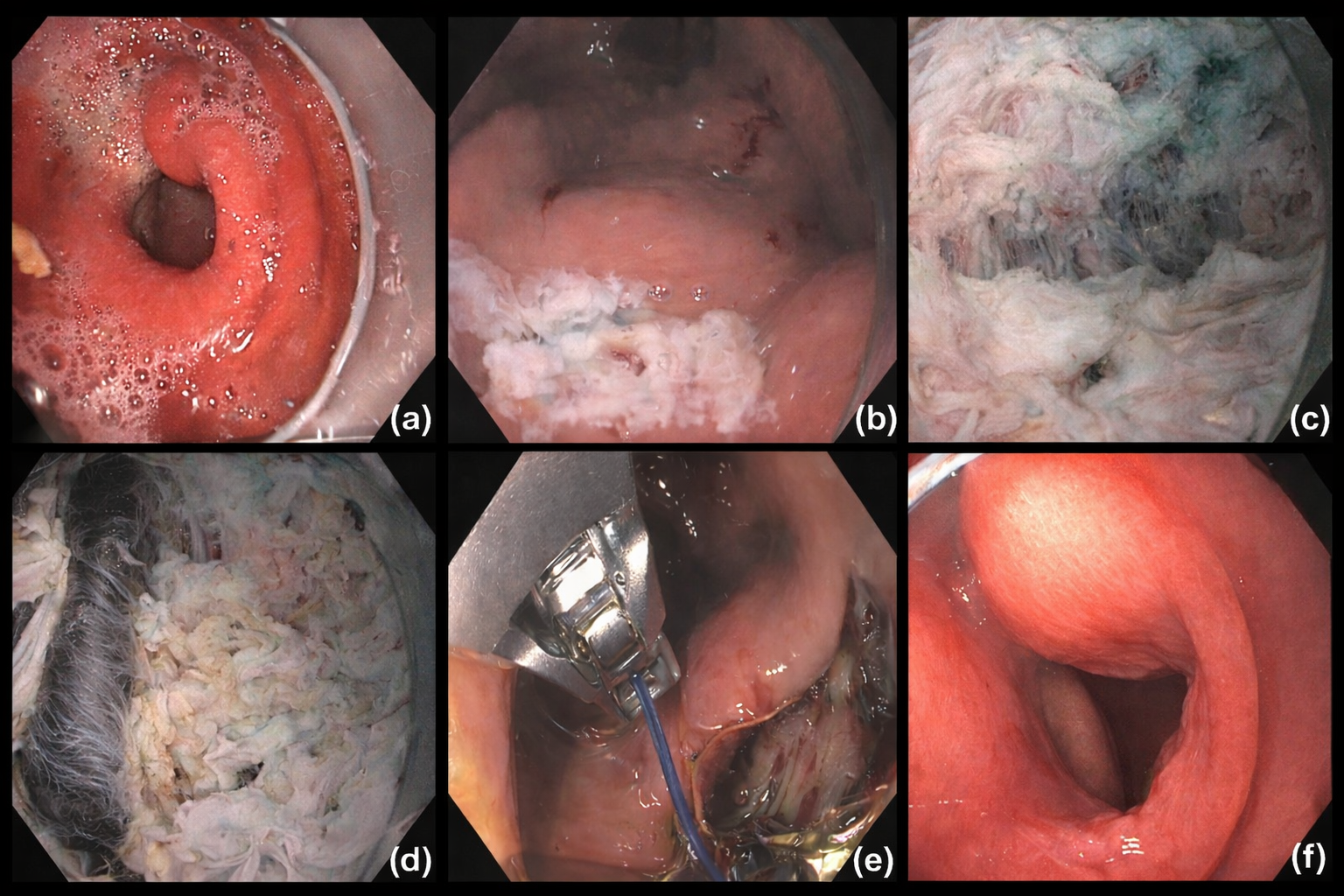

Supplement: Supplementary file 1 — FIGURE S1: Images highlighting the steps of the G‐POETS procedure. (a) Endoscopic identification of stricture. (b) Initial incision before insertion into the tunnel. (c) Stricture delineation. (d) Full‐thickness stricturotomy. (e) Submucosal tunnel closure. (f) Improvement in stricture opening. [file DEO2-6-e70290-s001.png]
